# Monocular Pose Estimation Method for Automatic Citrus Harvesting Using Semantic Segmentation and Rotating Target Detection

**DOI:** 10.3390/foods13142208

**Published:** 2024-07-13

**Authors:** Xu Xiao, Yaonan Wang, Yiming Jiang, Haotian Wu, Bing Zhou

**Affiliations:** 1 College of Electrical and Information Engineering, Hunan University, Changsha 410082, China; xiaoxu0913@hnu.edu.cn (X.X.); yaonan@hnu.com (Y.W.); ymjiang@hnu.edu.cn (Y.J.); wuhaotian@hnu.edu.cn (H.W.); 2 National Engineering Research Center for Robot Vision Perception and Control Technology, Hunan University, Changsha 410082, China; 3 College of Mechanical and Vehicle Engineering, Hunan University, Changsha 410082, China

**Keywords:** automatic harvesting, citrus fruit, monocular pose estimation, semantic segmentation, rotating target detection

## Abstract

The lack of spatial pose information and the low positioning accuracy of the picking target are the key factors affecting the picking function of citrus-picking robots. In this paper, a new method for automatic citrus fruit harvest is proposed, which uses semantic segmentation and rotating target detection to estimate the pose of a single culture. First, Faster R-CNN is used for grab detection to identify candidate grab frames. At the same time, the semantic segmentation network extracts the contour information of the citrus fruit to be harvested. Then, the capture frame with the highest confidence is selected for each target fruit using the semantic segmentation results, and the rough angle is estimated. The network uses image-processing technology and a camera-imaging model to further segment the mask image of the fruit and its epiphyllous branches and realize the fitting of contour, fruit centroid, and fruit minimum outer rectangular frame and three-dimensional boundary frame. The positional relationship of the citrus fruit to its epiphytic branches was used to estimate the three-dimensional pose of the citrus fruit. The effectiveness of the method was verified through citrus-planting experiments, and then field picking experiments were carried out in the natural environment of orchards. The results showed that the success rate of citrus fruit recognition and positioning was 93.6%, the average attitude estimation angle error was 7.9°, and the success rate of picking was 85.1%. The average picking time is 5.6 s, indicating that the robot can effectively perform intelligent picking operations.

## 1. Introduction

Citrus is one of the most important fruit tree genera in the world, with a planting area and yield ranking among the top in the world [1]. In China, the citrus industry has always been a pillar of rural economic development [2]. However, currently, citrus harvesting in China still relies on high-cost and high-intensity manual harvesting [3]. The insufficient supply of agricultural labor and the huge labor expenditure are seriously hindering the development of the industry [4]. Therefore, utilizing intelligent devices to automatically harvest fruits is of great significance for liberating productivity and improving production efficiency [5]. Due to the irregular position and angle of citrus growth, as well as the influence of branches and fruit occlusion, the harvesting conditions are complex and variable, making it difficult for harvesting robots to accurately identify and locate citrus fruits in complex orchard environments [6].

In recent years, robots have been widely used in industrial, agricultural, and other scenarios, and play an increasingly important role. As the most commonly used basic action of the robots, grasping is a very important ability of the robots to complete the task of picking and placing. However, because of unstructured farm environments and other uncertainties, it remains a huge challenge for robots to reliably grasp objects. The grasping task not only requires the robot to accurately identify the object in the scene but also needs the robot to accurately determine what the object is as a basic and key action. Grasping is the indispensable ability of the robot to perform the task of picking and placing. In an unstructured field environment, the challenge for a picking robot is not only to accurately identify the target fruit in the scene but also to precisely determine the location and orientation of the object. This requires robots to be able to achieve highly precise automation to adapt to complex farm environments. At present, machine vision detection technology is widely used in fruit detection tasks [7]. Sun proposed a multi-task learning model called FPENet to simultaneously locate the fruit navel point, predict the fruit rotation vector [8], and introduce hyperparameters into the loss function to achieve simultaneous convergence of multiple tasks [9]. A 2D image annotation tool was designed, and a citrus pose dataset was constructed, which is helpful for model training and algorithm evaluation [10]. Lin proposed a fruit detection and pose estimation method using low-cost red green blue depth (RGB-D) sensors [11]. Firstly, a state-of-the-art fully convolutional network is deployed to segment RGB images and output a binary image of fruits and branches [12]. Then, based on the fruit binary image and RGB-D depth image, Euclidean clustering is applied to group the point cloud into a set of individual fruits [13]. Subsequently, a multi-three-dimensional (3D) line segment detection method was developed to reconstruct the segmented branches [14]. Finally, its center position and nearest branch information are used to estimate the 3D pose of the fruit. Kim Taehyeong proposed a 2D pose estimation method for multiple rooted tomatoes based on a bottom-up approach [15]; first, detect all components related to tomato pose in an image, and then estimate the pose of each object. However, image-processing techniques based on color, shape, and texture require accurate feature information of the target fruit [16]. This method takes human pose estimation as the backbone and trains the model based on four key points defined in this study: tomato center [17], calyx [18], ionosphere [19], and branching points [20]. Rong Jiacheng aims to accurately identify the position of tomato fruits and estimate the grasping posture to improve the success rate and efficiency of robot harvesting [21]. A specially designed adsorption and clamping integrated robotic arm was proposed, and the optimal sorting algorithm and fruit nearest neighbor positioning algorithm were developed [22]. Directional grasping and sequential picking control strategies were designed to reduce the impact of dense fruits on the precise grasping of the robotic arm [23].

Overall, there is currently limited research on the target detection and pose estimation of citrus fruits in natural environments, and there have been no reports on mature intelligent harvesting technologies in natural orchard environments [24]. In an unstructured citrus orchard environment, a reliable and robust citrus fruit object detection algorithm is crucial for the development of harvesting robots. At the same time, fruit pose estimation is an important factor in guiding the end effector of the harvesting robot to approach the fruit for precise harvesting [25]. Therefore, this article proposes a monocular pose estimation method based on semantic segmentation and rotation object detection to address the high accuracy requirements and difficult pose estimation of citrus fruit target recognition and localization algorithms in natural orchard planting environments: Use Faster R-CNN for grabbing detection to obtain candidate grabbing boxes; combine with semantic segmentation networks to obtain contour information of citrus fruits; based on the semantic segmentation results, select the grabbing box with the highest confidence for each citrus fruit to be grabbed, and complete rough angle estimation in order to provide a technical basis for the development of citrus picking robots [26].

## 2. Related Work

### 2.1. Data Acquisition Platform

**Figure 1:**
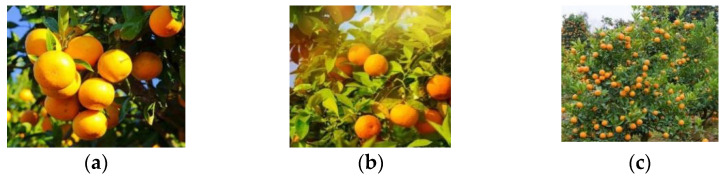
Example of citrus dataset. (**a**) Sunny day; (**b**) sunny backlight; (**c**) cloudy sky.

In this study, we need to pick citrus as the research object. All the pictures were taken in a natural citrus orchard at the Hunan Academy of Agricultural Sciences in Changsha, Hunan province. April 2024, China. The experimenters used Intel’s RealSense D435i (Intel Corporation, Santa Clara, CA, USA) as an image capture device. During the collection process, the experimenter controlled the camera at a distance of 0.3 to 0.6 m from the crown surface parallel to the fruit tree. The pixel resolution is fixed at 1280 × 720. The sample image is shown in Figure 1. In order to improve the robustness of model training, the data of the acquired original images were screened, and Labelme software 3.16.2 was used to manually label the images, and then the image data set was randomly divided into the training set, the test set, and the verification set at a ratio of 8/1/1.

### 2.2. Network Model Construction

**Figure 2:**
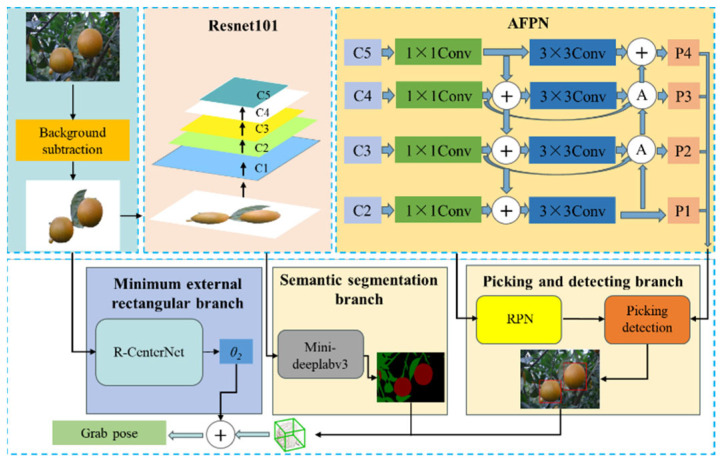
Citrus fruit picking pose-estimation network structure.

In this study, the two-dimensional grabbing rectangle generated in the RGB color images is used as the reference for the grabbing posture of the picking robot. Among them, the deflection angle of the detection frame is used as the angle when the picking robot works, and the pixel coordinate of the center point of the detection frame is converted into the three-dimensional coordinate value of the corresponding space point in the base coordinate system of the picking robot. In order to efficiently suppress background interference and the influence of irrelevant branches to obtain accurate segmentation images of citrus fruits and their attached branches, the background difference method based on RGB color information can be used to separate the citrus fruits to be captured from the natural background of the orchard after obtaining the background of the application scene, and the suppression of the background may introduce noise points in the outline of the object [27]. The effect of some noise points should be removed by morphological operation. However, this method does not suppress the effect of the shadow of the object to be grabbed in the scene. Therefore, the overall architecture of the network can be divided into two stages in this study, as shown in Figure 2. Stage 1: First, the capture detection algorithm Faster R-CNN is used to obtain the initial capture frame. Then, using the obtained results of a series of initial grasping frames and semantic segmentation, the outline information of the citrus fruits to be grabbed and the geometric information of initial grasping frames were integrated The grasping position of each citrus fruit to be grabbed was selected on the object, the frame with the highest confidence was taken as the initial grasping frame, and the rough angle estimation was completed. Stage 2: The angle prediction branch is used to obtain a fine grasp angle of citrus fruit to supervise the deflection angle of the grasp frame in stage 1.

(1) Faster R-CNN model

**Figure 3:**
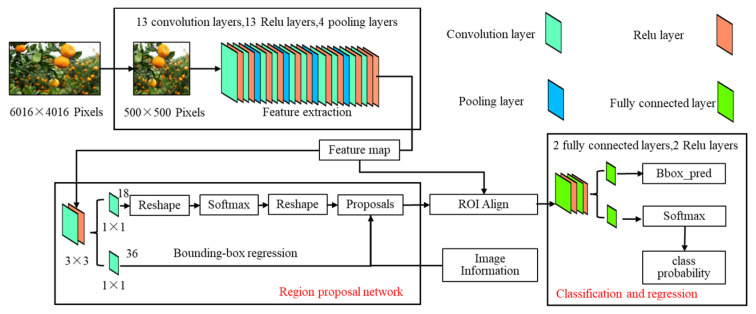
Framework diagram of Faster R-CNN.

In order to efficiently suppress the influence of background interference and irrelevant branches and obtain accurate segmentation images of citrus fruits and their attached branches, Resnet101 was used as the backbone network in the Faster R-CNN model to extract features. R-CNN, which stands for Region-CNN, was the first algorithm to successfully apply deep learning to object detection. The Faster R-CNN model consists of a backbone network, a regional suggestion network, and a detection head. Extract the features of the input image based on the backbone network and generate a feature map C1, C2, C3, C4, and C5. The feature pyramid network receives the feature graph {C2, C3, C4, C5} output by the backbone network, and fuses the feature graph of different levels to generate P1, P2, P3, and P4. In order to reduce the impact of citrus fruit features disappearing with the deepening of the network, the feature pyramid network adopts AFPN, which is an attention-based top-down and bottom-up network structure that can achieve multi-scale feature fusion. The regional generation network uses the feature map generated by AFPN to generate a series of grab candidate frames and output them to the picking detection head. The deflection angle of the picking detection head to the grab frame is classified as 18, while the pixel coordinates of the center point of the grab frame and the width and height of the grab frame are generated by regression operation. The network structure of Faster R-CNN is shown in Figure 3.

(2) FPNs (Feature Pyramid Networks)

The low-dimensional feature map of FPN mainly contains the local location feature information of objects such as edge lines but lacks sufficient semantic information. However, high-dimensional feature maps mainly contain global semantic information such as details and contours, but the position information is rough [28]. Usually, rich high-dimensional semantic information can obtain better detection results. Therefore, FPNs fuse the high-level features of low-resolution high-semantic information with the low-level features of high-resolution low-semantic information, so that the feature maps at each level of the pyramid can contain rich semantic information. However, location information is very important for picking detection, so this study added a bottom-up structure on the basis of an FPN, and added low-dimensional features to high-dimensional decision features, so as to obtain more accurate location information of citrus fruits. An attention feature pyramid network (AFPN) is proposed in this paper, which is a top-down and bottom-up structure based on a self-attention mechanism. The structure of the AFPN is shown in Figure 3, and the A in AFPN is the cosine non-local attention module. In AFPNs, the attention module and bottom-up structure are used to capture fine information of different scales in the image and achieve multi-scale feature fusion, so that all layers of the pyramid share similar semantic features and accurate location information, so as to improve the accuracy of picking detection.

(3) Semantic segmentation module

**Figure 4:**
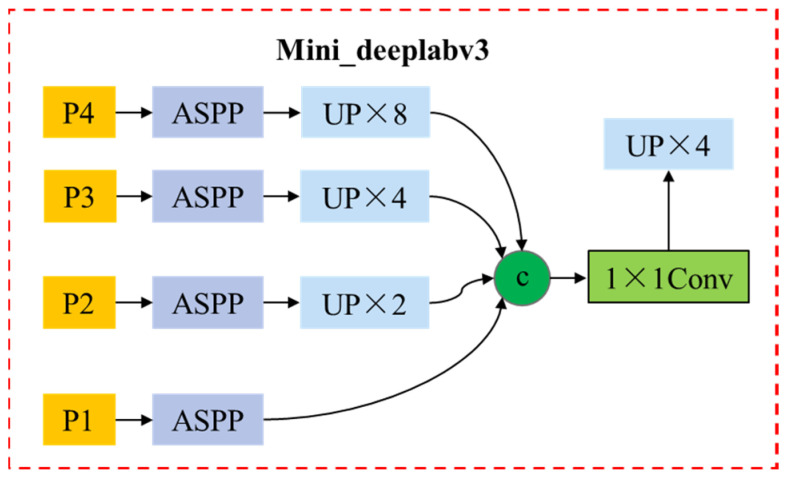
Mini deelabv3 Network structure.

Because the picking robot does not take into account the contour information of the citrus fruit to be picked in the picking detection of the target citrus fruit, it uses the detection frame of the target fruit to select a picking frame with the intersection and the highest confidence from a series of picking frames as the optimal picking frame, without considering whether the picking location is on the object [29]. Semantic segmentation can obtain the contour information of citrus fruits to be grabbed in the natural scene of the orchard. Therefore, the frame with the highest grasping position and the highest confidence can be selected from a series of grasping frames using the contour information of citrus fruits obtained by semantic segmentation as the grasping frame with the optimal grasping position. The semantic segmentation network structure of this paper is shown in Figure 4. The specific process of the semantic segmentation branch is divided into three steps. First, the semantic segmentation module receives the output of the Attention Feature Pyramid Network(AFPN) as input and sends it to Atrous Spatial Pyramid Pooling (ASPP) to obtain the outline information of the object to be grasped on the workbench [30]. Then, from a series of grasping frames generated from Faster R-CNN by using the outline information of objects to be grasped on the workbench, the frame with the highest confidence is selected for each object to be grasped as the initial grasping frame, and the rough angle estimation is completed.

(4) Citrus fruit pose estimation module

**Figure 5:**
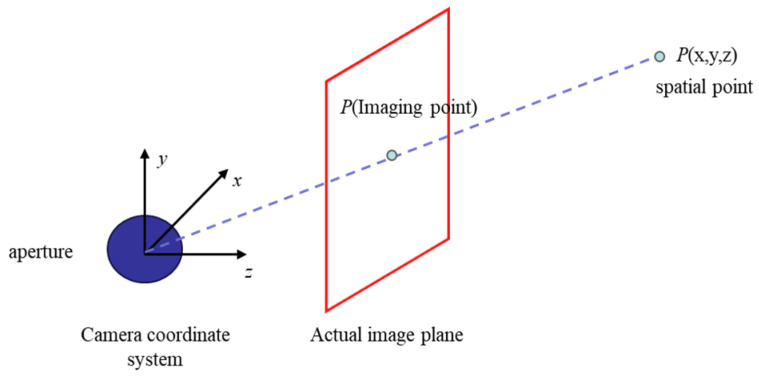
Pinhole imaging model of citrus fruit in natural environment.

The fruit’s spatial position and posture information can be determined based on the three-dimensional positioning of the citrus fruit’s three-dimensional boundary box, and the acquisition of the fruit’s three-dimensional frame needs to map the fruit from the three-dimensional space to the two-dimensional space [31]. The relationship between the actual three-dimensional space points and the actual imaging plane is usually calculated through the pinhole imaging principle. As shown in Figure 5, each pixel in the image is the intersection point between the aperture, the actual space point line, and the imaging plane, which are used to realize the imaging of the three-dimensional space point to the two-dimensional image.

**Figure 6:**
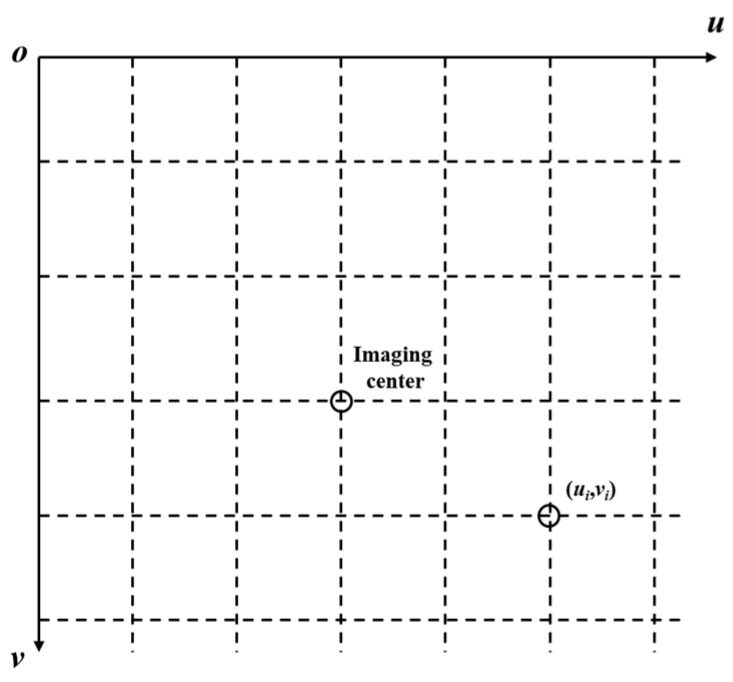
Pixel coordinate system.

**Figure 7:**
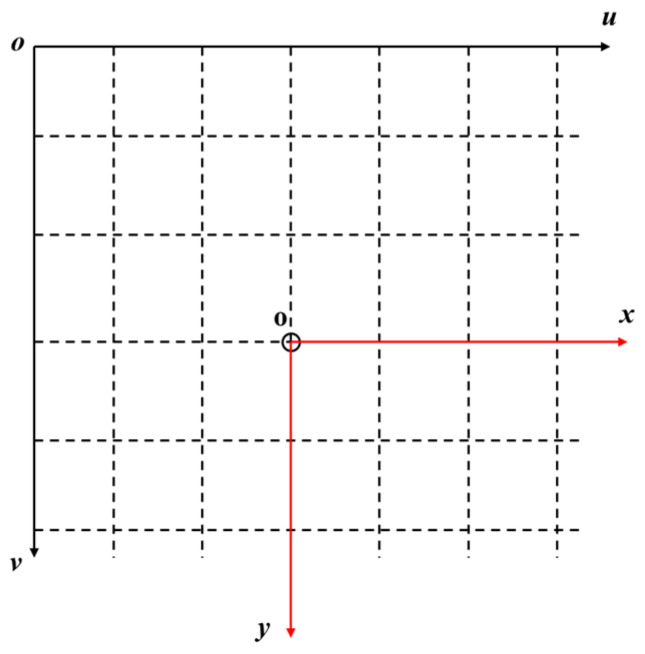
Image coordinate system.

In practical applications, the image captured by the camera and the data read are calculated by pixel points, usually based on the uov plane in the pixel coordinate system, as shown in Figure 6. Therefore, pixel coordinates need to be converted into image coordinates corresponding to two-dimensional space, and the image coordinate system is shown in Figure 7. Set the size of each pixel as *dx* and *dy*, and the pixel corresponding to the origin o of the image coordinate system is (*u*_0_, *v*_0_). By giving any specific pixel coordinate (*u*, *v*), its image coordinate (*x*, *y*) can be calculated. The calculation formula is as follows:

(1) $$\left[\begin{array}{c} x\\ y\\ 1 \end{array}\right]=\left[\begin{array}{ccc} dx & 0 & -u_{0}dx\\ 0 & dy & -v_{0}dx\\ 0 & 0 & 1 \end{array}\right]\left[\begin{array}{c} u\\ v\\ 1 \end{array}\right]$$

Furthermore, the spatial depth information obtained can realize the conversion of the two-dimensional image to three-dimensional spatial coordinates. According to the spatial calculation model of the camera, the joint vertical (1) can be obtained as follows:

(2) $$\left[\begin{array}{c} x_{p}\\ y_{p}\\ z_{p} \end{array}\right]=\left[\begin{array}{ccc} \frac{z_{p}}{f} & 0 & 0\\ 0 & \frac{z_{p}}{f} & 0\\ 0 & 0 & z_{p} \end{array}\right]\left[\begin{array}{ccc} dx & 0 & -u_{0}dx\\ 0 & dy & -v_{0}dx\\ 0 & 0 & 1 \end{array}\right]\left[\begin{array}{c} u\\ v\\ 1 \end{array}\right]$$

where f is the focal length of the camera; (*x_p_*, *y_p_*, *z_p_*) is the three-dimensional space coordinate of point P; *z_p_* is the depth of the actual space point 0P in the camera coordinate system, so as to calculate the space coordinates of the actual space point in the camera coordinate system.

The focal length of the camera in the x and y axes is defined as *f_x_* and *f_y_*, respectively, and Equation (2) is simplified to obtain the relationship between pixel coordinates and spatial three-dimensional coordinates as follows:

(3) $$\left[\begin{array}{c} x_{p}\\ y_{p}\\ z_{p} \end{array}\right]=z_{p}\left[\begin{array}{ccc} \frac{dx}{f_{x}} & 0 & \frac{-u_{0}dx}{f_{x}}\\ 0 & \frac{dy}{f_{y}} & \frac{-v_{0}dx}{f_{y}}\\ 0 & 0 & 1 \end{array}\right]\left[\begin{array}{c} u\\ v\\ 1 \end{array}\right]$$

To realize the one-to-one mapping between each pixel in the image and the actual three-dimensional space point. Based on the above principles, the three-dimensional boundary frame of the citrus fruit is fitted, and then the three-dimensional pose information of the citrus fruit is obtained.

The detected target position and attitude information can usually be determined by a 3D bounding box. P1–P8 are the 8 vertices of the citrus fruit 3D boundary box with coordinates (*X_i_*, *Y_i_*, *Z_i_*) (*i* = 1, 2, …, 8). Its coordinates can be obtained from the relative geometric position from *P_i_* to *Q_o_*, for example, the three-dimensional coordinates of point *P*_1_ are as follows:

$$X_{1}=X_{q}+\frac{dx_{c}}{2}$$

(4) $$Y_{1}=Y_{q}+\frac{dy_{c}}{2}$$

$$Z_{1}=Z_{q}+dz_{c}$$

## 3. Experimental Results and Analysis

### 3.1. Grab Data Set

In this paper, the network realizes the training and testing of the capture pose detection task under the Pytorch deep learning framework, which mainly relies on OpenCV 3.4.11, Numpy, matlibplot, and so on. The proposed algorithm runs in the Ubuntu 20.04 operating system with Intel Xeon E5700 V4@2.10 GHz CPU, 64 GB RAM, and NVIDIA GeForce RTX 2080 GPU (Intel Corporation). CUDA and cuDNN versions are 10.1 and 9.6.5, respectively. The average crossover ratio (mIoU) and average pixel accuracy (mPA) were used as evaluation indexes to evaluate the performance of the citrus segmentation model, and the angle error between the estimated and actual growth pose was used as an evaluation index to evaluate the evaluation effect of citrus pose estimation.

### 3.2. Citrus Image Segmentation Experiment

Compared with the Masked Fusion, FFB6D, PR-GCN, and PVN3D networks on the test set images, Table 1 shows that the performance of the AFPN model proposed in this paper is significantly superior to other four network models for image detection and segmentation of citrus fruits and its attached and growing branches. The average crossover ratio of fruit image segmentation is 89.96%, and the average pixel accuracy is 95.67%. The average crossover ratio of attached and growing branches image segmentation is 78.62%, and the average pixel accuracy is 75.62%. Although several models have a good segmentation effect on citrus fruit images, due to the difficulty of the image segmentation of citrus fruits attached to growing branches, PR-GCN and PVN3D are prone to mis-segmentation and image mask loss, and the contours of citrus branches are not smooth enough. The results of image segmentation using AFPN are better than those of the other four models.

### 3.3. Estimation of Citrus Fruit Growth Posture

**Figure 8:**
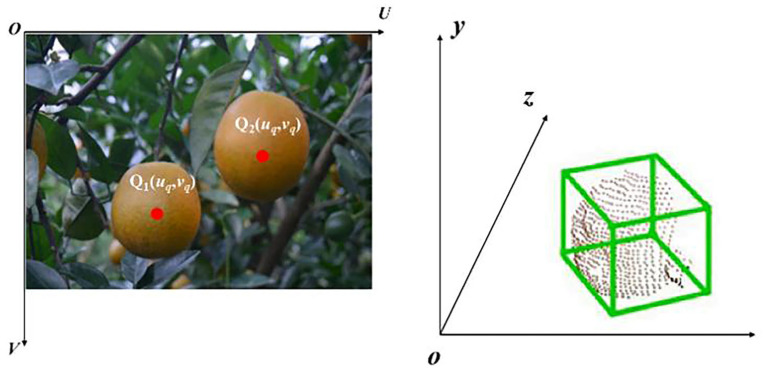
Examples of citrus 2D and 3D location information.

**Figure 9:**
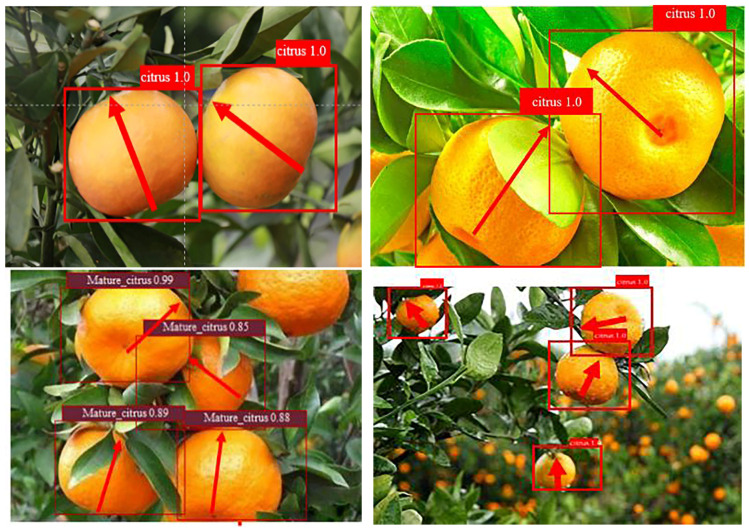
Citrus pose estimation vector diagram.

Firstly, the proposed algorithm was tested on the test set for citrus growth pose estimation, so as to test the stability of the algorithm and calculate the angle between the estimated growth pose and the actual growth pose (manually marked) to measure the citrus pose estimation error. The smaller the angle, the smaller the error, and the larger the angle, the larger the error. The angle *θ_i_* between the estimated growth attitude and the actual growth attitude is as follows:

$$\theta_{i}=arccos\frac{\tilde{q_{i}}q_{i}}{\left|\tilde{q_{i}}\right|\left|q_{i}\right|}$$

where is the estimated attitude of citrus; is the actual growth posture of citrus. The growth attitude estimation results of some citrus fruits measured on site are shown in Figure 8. In the figure, the yellow dot is the fruit growth point, the direction of the red arrow is the attitude estimated by the algorithm, and the direction of the black arrow is the real attitude. It can be seen that the angle error of this algorithm is small, and it has a good attitude estimation effect. At the same time, some attitude estimation failures also occurred in this study, as shown in Figure 9. The main reasons for the large error in attitude estimation are as follows: (1) When the citrus branches grow luxuriant, the image segmentation of the fruit-growing branches is easily mis-segmented, resulting in difficulty in determining the positional relationship between the fruit and its epiphytic branches. (2) In this algorithm, the junction point of citrus and its attached growing branches is defined as the fruit growth point, and it is defined as the starting point of the fruit pose estimation vector. Although this point is the closest point to the fruit centroid on the contours of the growing branches, it may not be the starting point of the best pose estimation vector.

**Figure 10:**
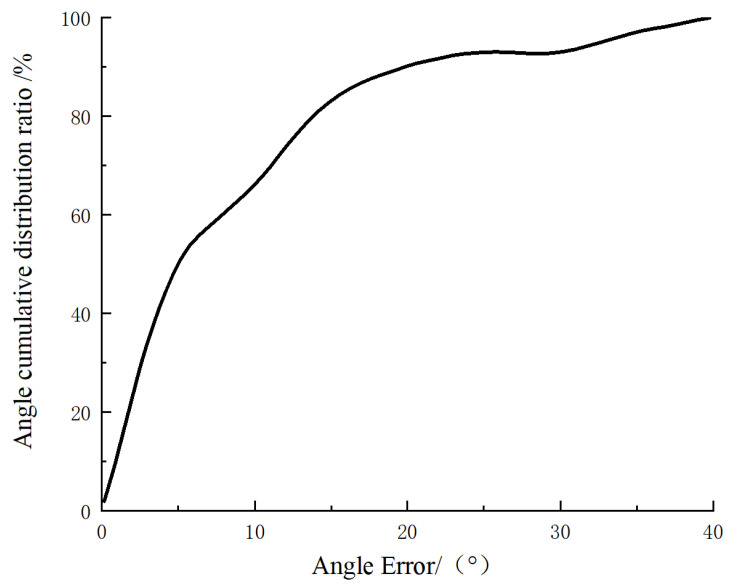
Proportion of cumulative angular error distribution.

The cumulative distribution ratio of angle errors is shown in Figure 10. The proportion of angle errors within 10° is 66.6%, the proportion of angle errors within 20° is 90%, the average of all angle errors is 8.8°, and the standard deviation is 7.2°.

### 3.4. Field Picking Experiment

**Figure 11:**
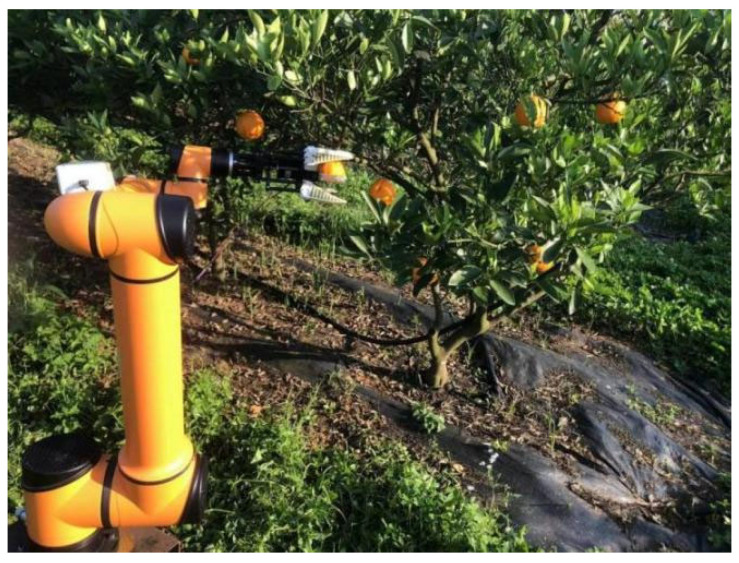
Field test environment of citrus picking robot.

The harvesting experiment was carried out in the citrus orchard of the Changsha Academy of Agricultural Sciences in the Hunan Province, and the branches of the fruit grew more luxuriant. The test time was from 1 PM to 6 PM, and the fruit trees used in the test were of the “Dafen No. 4” variety. The combination of the depth camera RealSense D455, the robotic arm, the end effector, and the tracked moving chassis constitutes the citrus-picking robot, as shown in Figure 11.

**Figure 12:**
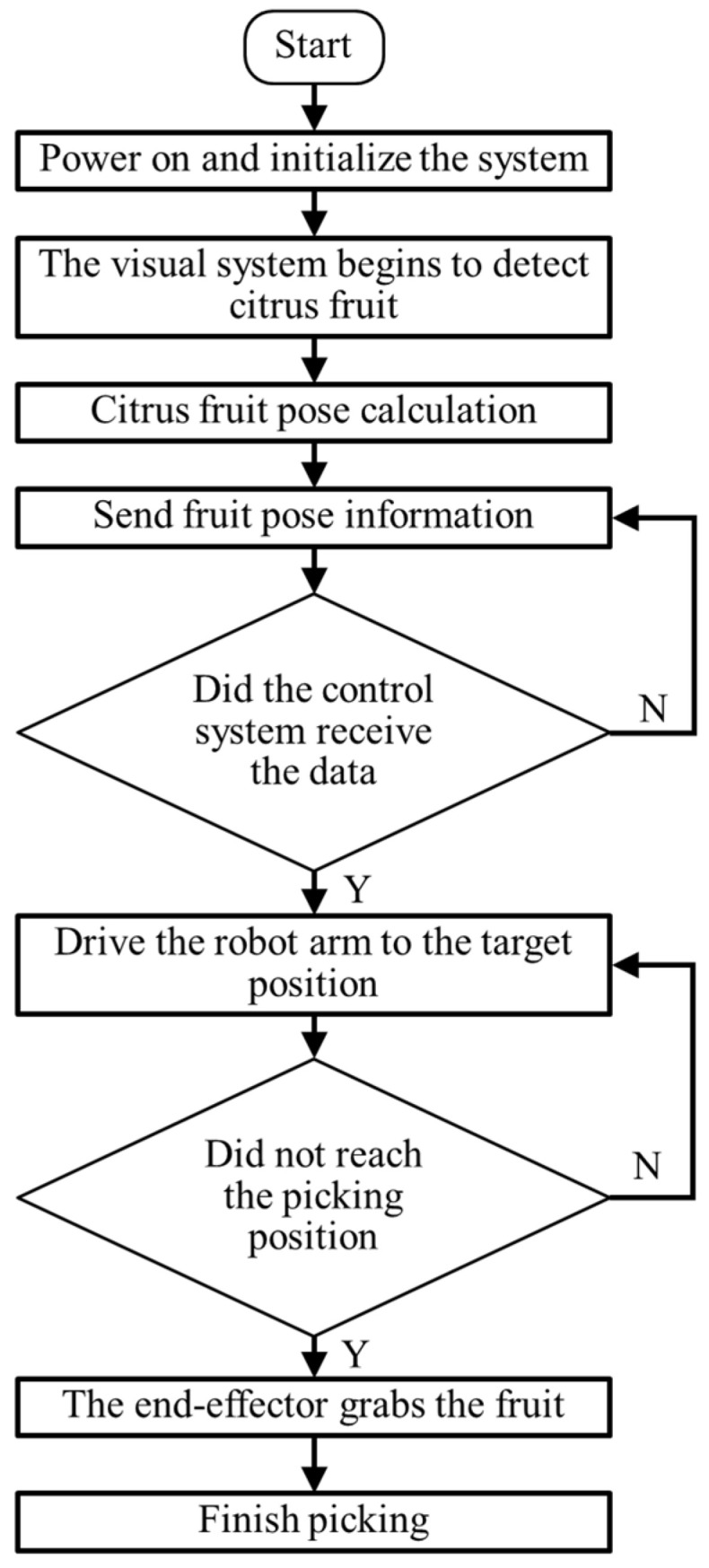
Robot flow chart.

The workflow of the citrus-picking robot is shown in Figure 12. After the picking robot was energized and initialized, it began to move along the planting line in the citrus orchard. When a ripe red citrus fruit is detected within the field of view set by the robot camera, the robot stops moving and begins to calculate the pose of the fruit and sends the pose information to the robot control module. After path planning is completed, the control command is sent to control the movement of the robotic arm to pick the fruit. If the branches seriously block the fruit, the citrus fruit picking will not be set up for the time being, and the obstacle avoidance picking research will be carried out instead. After the picking robot’s arm reaches the target position according to the attitude information of the citrus, the end effector grabs the citrus and rotates to pick it up. At the same time, there are some examples of fruit-picking failure in this experiment. The main reasons for the failure are the error of the attitude estimation angle and the instability of the mechanical claw. The picking test results are shown in Table 2: The recognition success rate of the citrus fruit picking robot arm in the natural field environment is 93.6%, and the average angle error of attitude estimation is 7.9°. The harvest was successful 1648 times, with a success rate of 85.1%. The average picking time was 5.6 s, indicating that the robot could basically complete the intelligent picking operation. During the picking test, locating the citrus fruits was unsuccessful mainly because the location of citrus fruits was beyond the picking range of the end effector, and the motion parameters of the robotic arm joints would produce errors, affecting the motion accuracy of the robotic arm, and leading to the picking failure.

## 4. Conclusions

Aiming at the pose detection task of citrus-fruit-picking robots, a new pose estimation method based on semantic segmentation and rotating target detection was proposed. First, Faster R-CNN was used for capture detection to identify candidate frames. Meanwhile, a semantic segmentation network was used to extract the contour information of citrus fruits. Then, the capture frame with the highest confidence was selected for each target fruit by semantic segmentation, which was convenient for rough angle estimation. Finally, field performance tests were carried out, and the success rate of citrus fruit identification and positioning was 93.6%, the average attitude estimation angle error was 7.9°, and the success rate of picking was 85.1%. The average picking time was 5.6 s, indicating that the robot can effectively perform intelligent picking operations. Specific conclusions are as follows:

(1) Aiming at the citrus-picking task of a citrus-picking robot, a single culture pose estimation method based on a semantic segmentation network and rotating target detection is proposed. In the upstream network comparison experiment of the data set constructed by us, the accuracy of the network capture detection proposed in this paper can reach 95.67%.
(2) The citrus picking robot developed based on the algorithm in this paper can accurately reach the target position and pick according to the control instructions. The success rate of picking in the natural environment of the orchard was 85.1%, and the average picking time was 5.6 s, which could meet the requirements for the mechanical picking of citrus.
(3) Judging from the failure of the grasping experiment, the influence of the centroid of citrus fruit and the friction between the end effector and the fruit can be considered in further picking and detection research, which is the focus of the future improvement of the citrus fruit positioning and grasping algorithm.

## Figures and Tables

**Table 1:** Comparison of Different Models for Sub-Image-Recognition Results.

Network Model	The Average Blending Ratio of Fruits	Average Pixel Accuracy of the Fruit	Average Crossover Ratio of Growing Branches	Average Pixel Accuracy of Growing Branches
AFPN	89.96	95.67	78.62	75.62
Masked Fusion	87.94	91.45	75.21	70.53
FFB6D	85.12	92.88	72.44	69.52
PR-GCN	81.08	88.73	78.54	73.39
PVN3D	85.48	88.90	72.25	74.54

**Table 2:** Field performance test results of picking robots.

	Number of Pick Attempts	Number of Successful Locations	Number of Picking Successes	Positioning Success Rate	Picking Success Rate
1	200	187	166	93.5	83
2	200	179	158	89.5	79
3	200	169	160	84.5	80
4	200	174	166	87	83
5	200	171	162	85.5	81
6	200	159	150	79.5	75
7	200	162	160	81	80
8	200	184	179	92	89.5
9	200	188	181	94	90.5
10	200	170	166	85	83
Total	2000	1743	1648	87.15	82.4

## Data Availability

The original contributions presented in the study are included in the article, further inquiries can be directed to the corresponding author.
